# Hemp Seed Oil and Oilseed Radish Oil as New Sources of Raw Materials for the Synthesis of Bio-Polyols for Open-Cell Polyurethane Foams

**DOI:** 10.3390/ma15248891

**Published:** 2022-12-13

**Authors:** Krzysztof Polaczek, Maria Kurańska

**Affiliations:** Department of Chemistry and Technology of Polymers, Cracow University of Technology, Warszawska 24, 31-155 Cracow, Poland

**Keywords:** open-cell foam, PUR, polyurethane, bio-polyol, hemp seed oil, used cooking oil, oilseed radish oil, rapeseed oil, epoxidation

## Abstract

We report on the development of open-cell polyurethane foams based on bio-polyols from vegetable oils: hemp seed oil, oilseed radish oil, rapeseed oil and used rapeseed cooking oil. The crude oils were pressed from seeds and subjected to an optimal solvent-free epoxidation process. Bio-polyols were obtained by a ring-opening reaction using diethylene glycol and tetrafluoroboric acid as catalysts. The resultant foams were analysed in terms of their apparent density, thermal conductivity coefficient, mechanical strength, closed cell content, short-term water absorption and water vapour permeability, while their morphology was examined using scanning electron microscopy. It was found that regardless of the properties of the oils, especially the content of unsaturated bonds, it was possible to obtain bio-polyols with very similar properties. The foams were characterized by apparent densities ranging from 11.2 to 12.1 kg/m^3^, thermal conductivity of <39 mW/m∙K, open cell contents of >97% and high water vapour permeability.

## 1. Introduction

Open-cell semi-rigid polyurethane (PUR) foams are used as thermal insulation materials. Their features are low thermal conductivity coefficient (36–42 mW/m∙K), low apparent density (8–15 kg/m^3^), high water vapour permeability and resistance to mould and mildew. Open-cell PUR foams are sprayed directly onto an uninsulated surface using a high-pressure application device [1,2,3]. The main application of open-cell spray PUR foams is the thermal insulation of attics, ceilings and interior walls [4].

PUR materials, including PUR foams, are mainly synthesized using raw materials of petrochemical origin. The global market for polyols was approximately USD 26 billion in 2019 and is expected to have grown to USD 34 billion by 2024 [5]. Increasing the sustainability of PUR through the use of renewable or waste-based raw materials has been a topic of research for years. Currently, a significant shift toward bio-based polyurethanes is also being observed across the PUR industry [5]. 

Renewable raw materials such as vegetable oils are mainly used to synthesize bio-based PUR [6,7], although other raw materials such as microalgae oils, lignin and polysaccharides are also being studied [5,8]. Waste raw materials such as tall oil (a waste from the cellulose industry) [9], waste poly(ethylene terephthalate) and used cooking oils are also being intensively studied [10,11].

The most common vegetable oils used to synthesize bio-polyols are rapeseed oil, sunflower oil, palm oil and soybean oil [5,6]. However, edible oils are important components of the human diet and their use in the chemical industry is controversial. Waste oils and non-edible oils can be alternatives to edible oils in chemical syntheses [12,13]. 

A non-edible oil used in the chemical industry to synthesize bio-polyols is castor oil. Unmodified castor oil contains hydroxyl groups in its structure and so can react with isocyanate groups to form a urethane bond. Disadvantages of castor oil are its low functionality and the presence of secondary hydroxyl groups that have lower reactivity compared to primary groups. Therefore, in order to improve its properties and broaden the applicability, it is necessary to subject the oil to chemical modifications, as in the case of other vegetable oils [6].

Used cooking oils (UCOs) also find application in the synthesis of bio-polyols. Studies have shown that changes in the chemical structure of UCOs have a negligible impact on the synthesis process and properties of bio-polyols [14]. A disadvantage of UCO in general can be its heterogeneous composition, which can be a mixture of many sorts of oils, as well as animal fats and impurities [15].

Another raw material with a great potential for use in chemical syntheses is non-edible oil from oilseed radish (OR) (*Raphanus sativus* (L.) var. *Oleiferus*) [16,17,18,19,20]. OR is characterized by a high content of erucic acid containing one double bond located in the 13th position along the fatty acid chain. The iodine value of crude OR is approximately 100–110 g I_2_/100 g, which is similar to that of rapeseed oil. Kim et al. [16] studied the epoxidation of OR with the use of peracetic acid. The conversion of OR to epoxidized oil was 69% and the selectivity of the process was 85%. It was found that epoxidation of oils with higher unsaturated bond contents (linseed oil, soybean oil and cottonseed oil) was characterized by higher conversion and selectivity. The use of OR as a raw material for the synthesis of bio-based PUR has not been described in the literature yet.

Another oilseed crop with high potential in the chemical industry is industrial hemp (Cannabis *sativa* L.). Nearly all parts of the hemp plant, including the fibres, seeds and inflorescence, find an industrial use [21]. Hemp seed oil (HSO) belongs to edible oils, but because of its low smoke point its main application is not in the food but in the cosmetics industry (shower gels, shampoos, hair and body balms, day and night creams and others) [22]. 

New varieties of industrial oil-type hemp are characterized by high seed yields with low growth of vegetative parts and a well-developed inflorescence, as well as a shorter growing period than in typical fibre hemp. The first registered variety of oil-type hemp is ″Henola″ grown at the Institute of Natural Fibres and Medicinal Plants (Poznań, Poland) with an average oil content of approximately 33 wt. % in dry seed weight [23]. HSO is characterized by a very high content of linoleic acid with two unsaturated bonds (C18:2, 55–60%), and linolenic acid with three unsaturated bonds (C18:3, 17–35%). The iodine value of HSO ranges from 140 to 175 g I_2_/100 g [24] and is higher than that of soybean oil (128–143 g I_2_/100 g), sunflower oil (110–143 g I_2_/100 g), rapeseed oil (110–126 g I_2_/100 g) and palm oil (44–58 g I_2_/100 g) [25]. Epoxidized HSOs are being investigated as raw materials for the synthesis of acrylated bio-polymers used mainly as a coating material for plastic, paper and wood [26], as well as for the synthesis of bio-based epoxy resins [24,27]. Surender et al. [28] synthesized hydroxyl derivatives of HSO by subjecting the oil to epoxidation and ring-opening reactions with various agents: water, ethanol and butanol. The products had hydroxyl values of 131, 120, 129 mgKOH/g, respectively, and were used to synthesize thermoplastic PUR elastomers. 

Here, we detail the development of open-cell PUR foams in which the polyol component was entirely bio-polyol synthesized from various oils: used cooking oil, rapeseed oil, oilseed radish oil and hemp seed oil. The selected raw materials are representatives of four groups of oils: edible oils, non-edible oils, waste oils and oils that are not a primary source of lipids. In the literature, there is no report on the synthesis of PUR foams containing bio-polyols from oil radish oil and hemp seed oil. 

**Figure 1:**
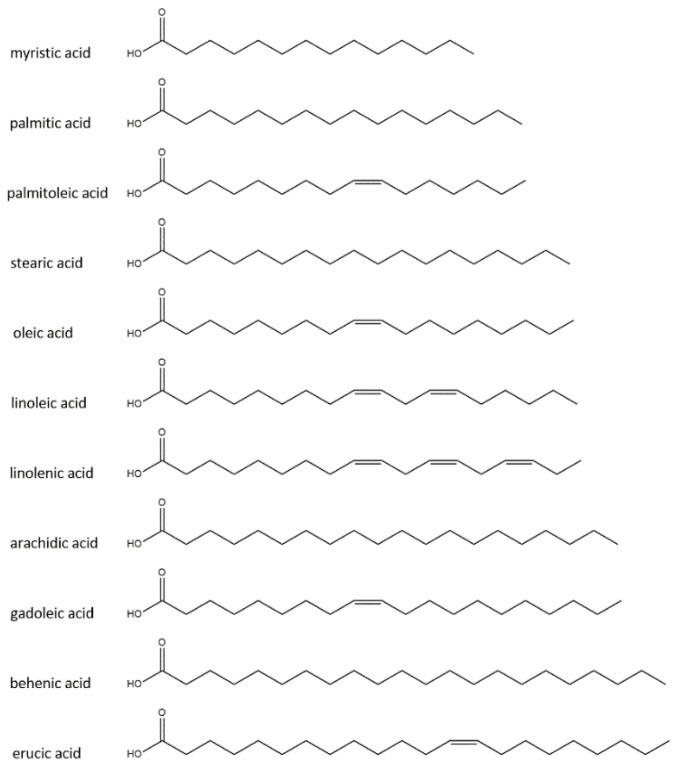
Chemical structure of fatty acids presents in the oils used.

Table 1 shows the fatty acid compositions of the oils used in the study based on the literature data. Figure 1 shows the chemical structure of fatty acids.

## 2. Materials and Methods

### 2.1. Plant Materials

**Figure 2:**
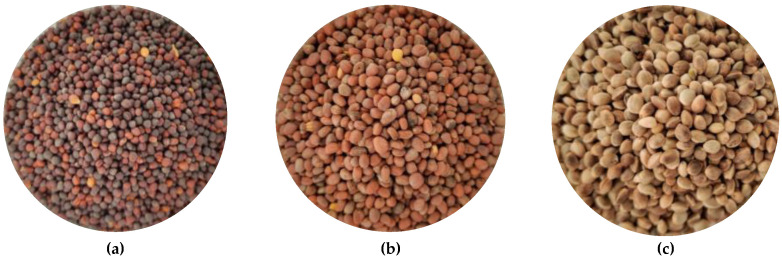
Oilseeds used: (**a**) rape; (**b**) oilseed radish; (**c**) hemp.

**Figure 3:**
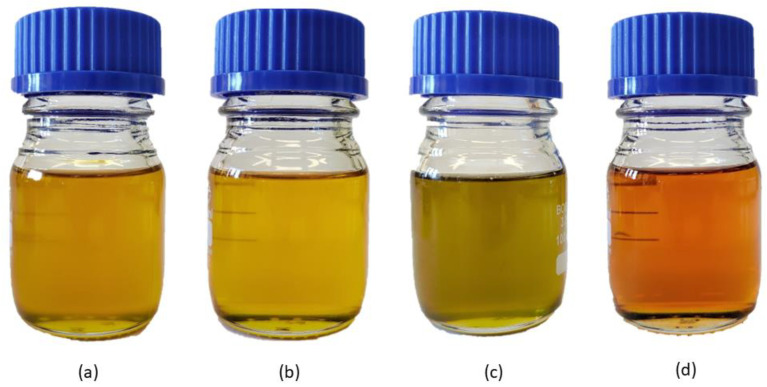
Oils used: (**a**) crude rapeseed oil; (**b**) crude oilseed radish oil; (**c**) crude hemp seed oil; (**d**) used cooking oil.

Seeds of oilseed radish (*Raphanus sativus* L. var. *oleiformis*) and rapeseed (*Brassica napus* L. var. *napus*) were purchased from FN Granum (Wodzierady, Poland); industrial hemp (*Cannabis sativa* L. var. *sativa*) was purchased from Nutrilla (Węgrzce, Poland). Used cooking oil (UCO) was supplied by local restaurants. Vegetable oils were extruded using a Wartmann (Tilburg, The Netherland) WM-2002OP miniature extruder. The oils were not subjected to any chemical refining process, only decanted from the sediment. Figure 2 shows the oilseeds used. Figure 3 shows the crude oils and UCO.

### 2.2. Reagents

Glacial acetic acid (GAA) (99.5 wt %), hydrogen peroxide (HP) (30 wt %) and diethylene glycol (DEG) were supplied by Avantor Performance Materials Poland (Gliwice, Poland). Ion exchange resin Amberlite^®^ IR 120 H and tetrafluoroboric acid (48% wt. % in water) were provided by Sigma-Aldrich (Darmstadt, Germany). Polymeric methylene-4,4′-diphenyl diisocyanate (pMDI) Ongronat^®^ 2100 was supplied by BorsodChem (Kazincbarcika, Hungary). Polycat^®^ 15 (non-emissive balanced amine catalyst), Polycat^®^ 140 (reactive catalyst with high selectivity towards the water-isocyanate blowing reaction), KOSMOS^®^ 19 (gelling catalyst), TEGOSTAB^®^ B 8870 (polysiloxane polyether surfactant used in high-water formulations), ORTEGOL^®^ 500 (cell-opener) and Dabco^®^ EM400 (emulsifier used in water-blown, low-density spray polyurethane foam) were supplied by Evonik Industries AG (Essen, Germany). TCPP (tris (1-chloro-2-propyl) phosphate) used as a flame retardant was purchased from Lanxess AG (Cologne, Germany).

### 2.3. Characterization of Oils and Their Derivatives

Vegetable oils, epoxidized oils and bio-polyols were subjected to analytical, physicochemical and spectroscopic analyses.

Iodine value (Ival) determining the content of unsaturated bonds was investigated in accordance with the PN-87/C-04281 standard and calculated by using Equation (1).

(1) $$I_{val}=\frac{\left(V_{b}-V_{s}\right)\cdot C_{t}\cdot 12.69}{m_{s}}\;\;g\;I_{2}/100g$$

where *V_b_* and *V_s_* are volumes in mL of sodium thiosulfate solution used for the blank and sample, respectively; *C_t_* is the concentration of sodium thiosulfate solution in mol/dm^3^; *m_s_* is the mass of the sample in g.

Hydroxyl value (Hval) was determined in accordance with the PN-C-89052-03:1993 standard. In this method, the hydroxyl groups undergo acetylation using acetic anhydride. The excess of the acetic anhydride is decomposed by a water addition and followed by titration using a solution of potassium hydroxide in the presence of thymolphthalein as an indicator. Hval was calculated by using Equation (2)

(2) $$H_{val}=\frac{\left(V_{b}-V_{s}\right)\cdot C_{KOH}\cdot 56.11}{m_{s}}+A_{val}\;\;\mathrm{mgKOH}/g$$

where *V_b_* and *V_s_* are volumes in ml of potassium hydroxide used for the blank and sample, respectively; *C_KOH_* is the concentration of potassium hydroxide solution in mol/dm^3^; *m_s_* is the mass of the sample in g. 

Acid value (*A_val_*) was determined in accordance with PN-ISO 660:2009 standard and calculated by using Equation (3)

(3) $$A_{val}=\frac{\left(V_{s}-V_{b}\right)\cdot C_{KOH}\cdot 56.11}{m_{s}}\;\mathrm{mgKOH}/g$$

where *V_b_* and *V_s_* are volumes in mL of potassium hydroxide used for the blank and sample, respectively; *C_KOH_* is the concentration of potassium hydroxide solution in mol/dm^3^; *m_s_* is the mass of the sample in g. Samples of approximately 10 g were used. 

Epoxy value (*E_val_*) was determined in accordance with the PN-87/C-89085/13 standard. The method involves a quantitative reaction of hydrochloric acid with epoxy group at room temperature and titration of the hydrochloric acid excess using a solution of sodium hydroxide in methanol in the presence of cresol red as an indicator. Eval was calculated by using Equation (4)

(4) $$E_{val}=\frac{\left(V_{b}-V_{s}\right)\cdot C_{NaOH}\cdot 100}{m_{s}\cdot 1000}\;\mathrm{mol}/100g$$

where *V_b_* and *V_s_* are volumes in ml of sodium hydroxide used for the blank and sample, respectively; *C_NaOH_* is the concentration of potassium hydroxide solution in mol/dm^3^; *m_s_* is the mass of the sample in g. Samples of approximately 0.3 g were used. 

Viscosity was determined using a Haake Viscotester 7 plus from Thermo Fisher Scientific (Waltham, MA, USA) at 25 °C (298 K). 

Water content (%H_2_O) was determined by Karl Fisher method in accordance with PN-81/C-04959 with the use of a TitroLine TA 05 plus device manufactured by SCHOTT Instruments GmbH (Meinz, Germany). 

The chemical structures were analysed using an FTIR spectrometer model Nicolet iS5 from Thermo Fisher Scientific (Waltham, MA, USA), equipped with an ATR (attenuated total reflection) accessory with a diamond crystal in the infrared range of 4000–500 cm^−1^. 

The number average molecular weight (Mn) and weight average molecular weight (Mw) were determined using gel permeation chromatography (GPC) analysis with the use of a chromatograph equipped with a refractometric detector. The analyses were performed at 25 °C. Tetrahydrofuran was used as an eluent and its flow rate was fixed at 1 mL/min. The functionality (f) refers to the number of OH groups per molecule was calculated following Formula (5):

(5) $$f=\frac{Hval\cdot Mn}{56110}$$

### 2.4. Characterization of PUR Foams

The apparent density of foam samples was calculated in accordance with the ISO 845:2006 standard as a ratio of the masses and volumes of samples. 

Thermal conductivity coefficient was measured with the use of a heat flow meter instrument Fox200 (TA Instruments, DE, USA) in accordance with the ISO 8301 standard. The foam samples with dimensions of 5 cm × 20 cm × 20 cm were placed in the apparatus. Heat flow was measured at plate temperatures of 0 and 20 °C. 

Compressive strength was measured at 10% deformation using a Zwick Roell testing machine (Zwick Roell Group, Ulm, Germany) according to the PN-EN 826:2013-07 standard. The mechanical properties of the foams were evaluated in a direction perpendicular to the foam growth direction. Eight cylindrical-shaped samples with a diameter of 40 mm and a height of 40 mm cut from the cores of two polyurethane foams were tested. 

The short-term water absorption test was carried out according to the PN-EN 1609:2013 standard.

Water vapour permeability (δ) and water vapour diffusion resistance factor (μ) were determined according to the PN-EN 12086:2013 standard. The following conditions were used in the test: 23 °C, 85% relative humidity, anhydrous calcium chloride was used as a desiccant. Six samples from each foam were tested. The weight of the test assembly was measured at regular 24 h intervals until the change in mass was constant within ± 5% of the mean value. 

The foam morphology was examined using a scanning electron microscope (SEM) Apreo 2 S LoVac (Thermo Fisher Scientific, Waltham, MA, USA) configured with a secondary electron detector in low vacuum mode and an acceleration voltage of 22 kV. The samples were coated with a 4 nm layer of carbon. Magnifications of 70 and 250 times were used. The analysis of the foam morphology (cell cross-section area) was performed using ImageJ software (U.S. National Institutes of Health, Bethesda, MD, USA, https://imagej.nih.gov/ij/, accessed on 12 December 2022, ver. 1.53 t) 

### 2.5. Synthesis of Epoxidized Vegetable Oils

The reaction setup consisted of a water bath in which four 1 dm^3^ round-bottom flasks equipped with identical stirrers set at 500 rpm, a thermometer and a reflux condenser were placed. Amounts of 250 g of oil and 37.5 g of Amberlite IR 120 H catalyst (15% by weight of oil) were added to the flasks and the bath temperature was set to 65 °C. After the set temperature was reached, 15 g of GAA and 133 g of HP were added. The amount of GAA and HP were calculated according to Equations (6) and (7), assuming that regardless of the oil used and its initial Ival, the final Eval was expected to be 0.2 mol/100 g.

(6) $$m_{GAA}=Ev_{exp}\times m_{oil}\times 0.5\times 60.05$$

(7) $$m_{HP}=\frac{Ev_{exp}\times m_{oil}\times 2\times 34.01}{30}$$

where *m*_*GAA*_ is the mass of glacial acetic acid [g], *m*_*HP*_ is the mass of hydrogen peroxide [g], *EV*_*exp*_ is the expected *Ev* value [mol/100 g], *m*_*oil*_ is the mass of the oil subjected to epoxidation [g].

The amounts of GAA and HP were 0.5 and 2 moles, respectively, for every 1 mol of unsaturated bonds subjected to the epoxidation reaction. The value of Eval of the epoxidized oils was selected based on previous studies [30], which showed that Eval above 0.2 mol/100 g led to a significant increase in the viscosity of the bio-polyol with a slight increase in the hydroxyl group content. In addition, reducing the use of acetic acid and hydrogen peroxide is beneficial from the ecological and economic point of view. The course of the reaction was monitored by periodically (every 20 min) measuring Eval. The effect of the chemical composition of the oils on the epoxidation process was studied by running the reactions for 9 h. Then, the epoxidation reactions of unrefined oils were carried out for 3 h and, in the case of UCO, for 4 h 20 min. After the reaction was completed, the oils were washed 4 times with 500 mL of warm water and distilled under reduced pressure.

### 2.6. Synthesis of Bio-Polyols

The syntheses of bio-polyols were carried out in a 0.5 dm^3^ reactor equipped with a heating mantle, a mechanical stirrer and a thermometer. Each time, 200 g of epoxidized oil was used. After the temperature of epoxidized oil reached 80 °C, a mixture of DEG (in stoichiometric amount to epoxy group content) and 0.4 g of tetrafluoroboric acid (0.2% by weight of the epoxidized oil) was added to the reactor while maintaining intensive mixing. The change in the epoxy value was monitored by taking samples every 10 min. The reaction was carried out until all the epoxy groups were reacted (titrated Eval = 0 mol/100 g).

### 2.7. Preparation of Polyurethane Foams

The PUR foams were obtained using a one-step two-component method. One of the components (A) consisted of bio-polyol, surfactants, catalysts, water and a flame retardant. The second component (B) consisted of isocyanate. The volume ratio of components was 1:1. The temperature of components A and B was 40 °C. Component A was mixed for 30 s with a mechanical stirrer and then component B was added and stirred for 3 s. The mixture was poured into a horizontal mould that provided free growth of the foam. The use of a mechanical stirrer affects the properties of foams but gives basic information on whether a given polyurethane system is suitable for industrial-scale testing with high-pressure spray machines. Samples were cut out 24 h after obtaining the foams. The formulation of the polyurethane systems is shown in Table 2. 

## 3. Results

### 3.1. Characteristics of the Oils 

Table 3 shows the properties of the extruded oils and UCO. Depending on the composition of fatty acids in triglyceride molecules (Table 1), the oils differ in Ival, which determines the content of unsaturated bonds in the molecules. The smallest Ival was observed in RO and UCO, which consist mainly of oleic acid with one double bond. A slightly higher Ival was found for OR, which consists mostly of erucic acid and in smaller amounts of oleic and gadoleic acids (each with one double bond), as well as linoleic and linolenic acids with two and three double bonds, respectively. The highest Ival was found for HSO containing mainly linoleic acid and linolenic acid. The Aval value determines the content of free fatty acids and is the highest for OR and HSO and the lowest for RO. For UCO, both the Ival and Aval values indicate the rate of oil degradation. Deep-fat frying decreases unsaturation while increases viscosity, as well as contents of free fatty acids and polymeric compounds [31]. 

### 3.2. Synthesis of Epoxidized Oils

**Figure 4:**
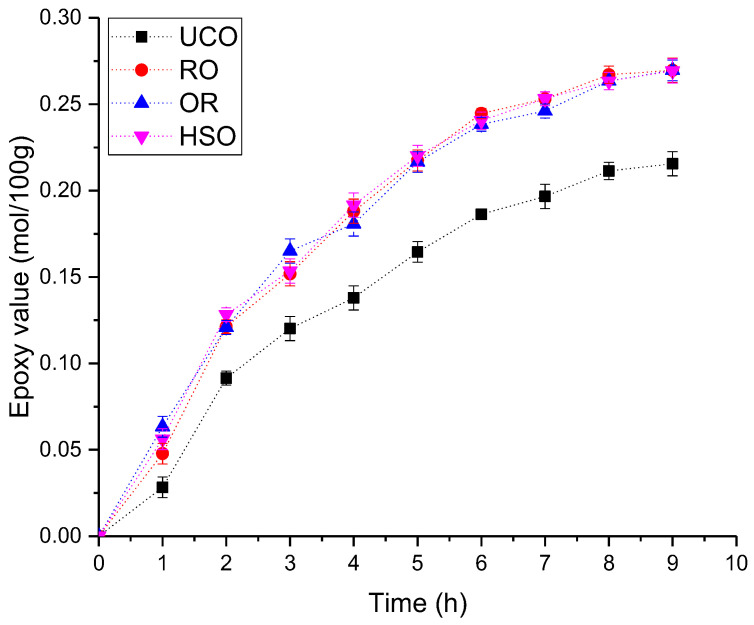
The change in Eval of the oils during the epoxidation process.

Vegetable oils were subjected to the epoxidation process. Figure 4 shows the change in Eval of the oils epoxidized for 9 h.

Unrefined oils (RO, OR, HSO) were characterized by a very similar course of epoxidation. The UCO epoxidation reaction was noticeably slower. After approximately 8 h, a flattening of the curve was observed, which can be explained as a result of the amount of the hydrogen peroxide and acetic acid used in relation to the double bond content (see Section 2.5) calculated relative to the assumed Eval of 0.2 mol/100 g rather than the initial Ival. According to the literature, the course of the epoxidation reaction is influenced by the fatty acid composition of an oil. The double bonds of oleic acids and linoleic acids in triglycerides are equally reactive, while the double bonds of linolenic acid are approximately three times more reactive toward epoxidation [32]. Additionally, free fatty acids can form peroxy fatty acids with H_2_O_2_ and act as an oxygen carrier to accelerate the epoxidation process [33]. However, the presence of fatty acids can also lead to excessive ring opening and the formation of estolides (oligomeric fatty acid esters containing secondary ester bonds on the alkyl backbone of the molecule) or other oligomers [34]. Crude oils contain approximately 2–5% of minor constituents including fatty alcohols, waxes, hydrocarbons, phenolic compounds, tocopherols, pigments, phospholipids, phosphorus and sulphur [35], as well as metals [36]. UCO is an oil that has undergone a refining process involving the removal of most of the impurities [37] which may have affected the epoxidation process. In the case of the reactions carried out in the presented study, the epoxidation rate could not be linked to either the fatty acid composition of the oils or their acid number, but most likely to the content of the impurities in unrefined oils. The epoxidation of oils under the test conditions could also be limited by the amount of acetic acid and hydrogen peroxide.

Table 4 shows the properties of the resulting epoxidized oils. The epoxidized oils were characterized by Eval in the range from 0.19 to 0.20 mol/100 g. The double bond residue is confirmed by the Ival of the epoxidized oils, ranging from approximately 41 g I_2_/100 g for UCO to 93 g I_2_/100 g for HSO. The side reactions taking place during epoxidation are reflected by the slightly higher viscosity of the epoxidized oils compared to the initial oils.

### 3.3. Synthesis of Bio-Polyols

Table 5 shows the properties of the bio-polyols obtained in the reactions. As expected, the bio-polyols were characterized by similar Hval. P_UCO and P_RO had slightly higher molecular weights and, consequently, viscosities compared to the other bio-polyols.

### 3.4. FTIR Spectroscopy Analysis

**Figure 5:**
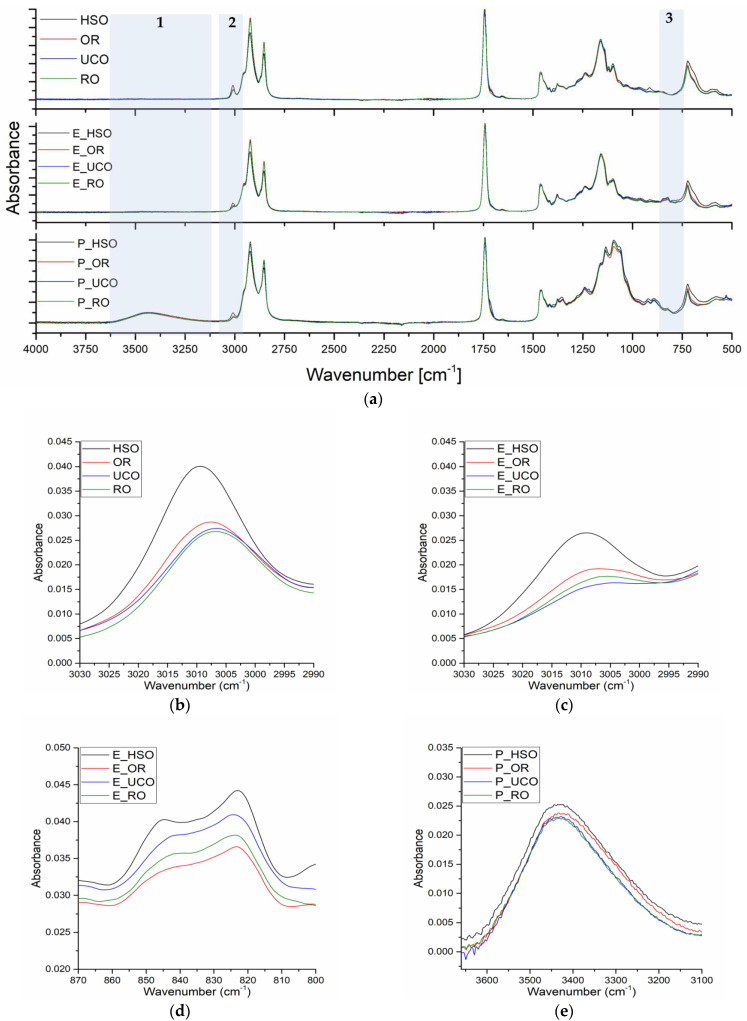
FTIR spectra: (**a**) overall spectra of oils, epoxidized oils and bio-polyols, (**b**) stretching vibrations of the cis-double bond (=C–H) of oils, (**c**) stretching vibrations of the cis-double bond (=C–H) of epoxidized oils, (**d**) oxirane ring vibrations, (**e**) hydroxyl group vibrations.

In spectra of the vegetable oils used in the reactions, bands with a maximum at 3009–3005 cm^−1^ originating from stretching vibration of =C-H groups are present. The intensity of these bands depends on the content of unsaturated bonds in the oil (Figure 5b). The presence of unsaturated bonds is also visible in the spectra of the epoxidized oils (Figure 5c). The peaks at 823 cm^−1^ originate from epoxy groups (Figure 5d). Figure 5e shows a close-up region of the hydroxyl group band with a maximum at 3433 cm^−1^. The slight differences in the intensity of these peaks visible in the figure confirm similar contents of hydroxyl groups in the final bio-polyols.

### 3.5. GPC Analysis

**Figure 6:**
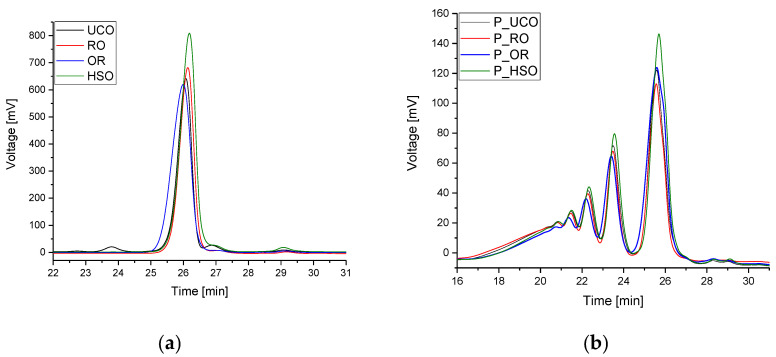
GPC chromatograms of oils (**a**) and bio-polyols (**b**).

Figure 6a shows GPC chromatograms of the vegetable oils used. The peaks with a retention time of 24–26 min originate from triglyceride molecules. In the case of UCO, a peak with a retention time of 23.7 min was also observed indicating dimers formed during the use of the oil, as well as a peak with a retention time of 27 min indicating diglycerides. The chromatograms of all oil samples show peaks with a retention time of 29.1 min indicating the presence of free fatty acids. Figure 6b shows GPC chromatograms of the bio-polyols. During the oxirane ring-opening reaction, many side reactions take place, including oligomerization reactions [38]. Peaks with retention times of approximately 26 min come from the hydroxyl derivative of triglyceride molecules. Peaks with retention times of 23.6, 22.3, 21.5 min come from dimers, trimers and tetramers of triglyceride molecules, respectively. The retention times ranging from 20.9 to 17.5 min indicate the presence of a mixture of oligomers.

### 3.6. Manufacturing of Open-Cell Polyurethane Foam

The resultant PUR foams were analysed in terms of their apparent density, thermal conductivity, compressive strength, open-cell content, water vapour permeability, water vapour diffusion factor and short-term water absorption. SEM images were taken to study the morphology of the PUR foams. The properties are given in Table 6.

The apparent densities of the foams had similar values and ranged from 11.2 for F_UCO and F_HSO to approximately 12.1 kg/m^3^ for F_OR and F_RO. The value of the apparent density of open-cell foams should be in the range of 8–15 kg/m^3^ [1]. For commercially available open-cell PUR foams containing only petrochemical polyols, the apparent density is typically 7–9 kg/m^3^ [2,39], and for foams based on bio-polyols 9.6–12.8 kg/m^3^ [3]. In general, a low apparent density is highly preferable as it offers lower material costs and load applied to insulated structures.

The thermal conductivity coefficient for the foams obtained ranging from 36.10 mW/m·K for F_RO to 38.75 mW/m·K for F_UCO represents typical values for open-cell foams comparable to commercial products [40,41] which on average have a thermal conductivity coefficient of approximately 36–38 mW/m·K.

Closed-cell content is a very important parameter for open-cell systems. If closed-cell content is high (>20%), the foams may be dimensionally unstable. All the foams obtained in our work had open-cell content above 95%, making them dimensionally stable and free from shrinkage.

An important advantage of open-cell foams is their high water vapour permeability, which prevents the formation of mould and mildew in insulated spaces. The maximum value of water vapour diffusion resistance factor μ of the foams obtained was approximately six, which makes them compliant with the standards for commercial foams. The vapour permeability of PUR foams is mainly affected by the content of closed cells. As the content of closed cells increases, the water vapour resistance factor increases (for closed-cell foams μ > 50) [1].

In our studies, the foams were characterized by very similar short-term water absorption of 0.18–0.25 kg/m^2^. This value is lower than for commercial foams, for which short-term water absorption values vary from 0.3 kg/m^2^ [40] to values of more than 1 kg/m^2^ [42]. Generally, foams with a higher content of closed cells have lower water absorbability [43]. In addition, an increase in the apparent density and a decrease in cell size decrease water absorption [44]. The value of water absorption is also affected by the type of polyols used. An addition of bio-polyols from vegetable oils to a PUR system results in the introduction of hydrophobic groups into the PUR chain, which causes a significant reduction in water absorption [45,46,47].

**Figure 7:**
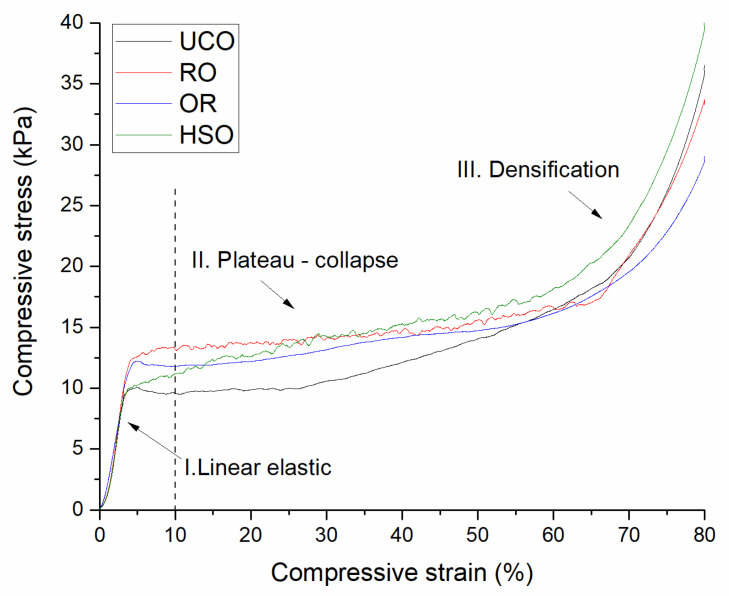
Stress–strain curves of the polyurethane foams showing the linear elastic, plateau-collapse and densification regimes.

Open-cell PUR foams are not designed to handle heavy loads, nor to strengthen an insulated structure. The compressive strength of the foams obtained in our experiment, ranging from 9.25 to 12.63 kPa, complies with the standards and is comparable to that of commercial foams [3]. Figure 7 presents the stress–strain curves of selected PUR foam samples showing three deformation regimes typical for cellular materials: linear elastic, plateau and densification. Linear elasticity is controlled by cell wall bending. The plateau region is associated with cell collapse. When most of the cells are completely collapsed, the cell walls touch each other and, with further compression, the material densifies giving the last region of rapidly increasing stress [48]. The point of 10% deformation in which the mechanical strength was tested for all the obtained foams was located in the collapse-plateau regime.

**Figure 8:**
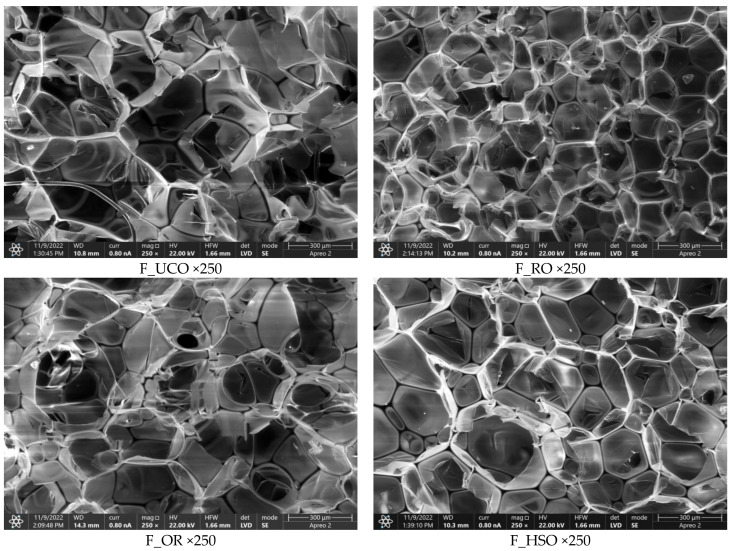
SEM images of the polyurethane foams at a magnification of 250×.

SEM images of the foams obtained in our work (Figure 8) made it possible to determine cell cross-section areas. The F_RO foam had the smallest cell size, which could also translate into its lowest thermal conductivity coefficient. Heat conduction in PUR foams occurs by convection, conduction of the gaseous phase, conduction of the solid polymer and radiation [49]. With no gas trapped in the cells of open-cell foams, the morphology of the cells has a major impact on their thermal properties. In general, as cell size decreases, free gas convection and heat flow through radiation (contributing a small share to the total heat flow in open-cell PUR foams) decrease, and as apparent density increases, heat transport through the solid phase increases [50].

**Figure 9:**
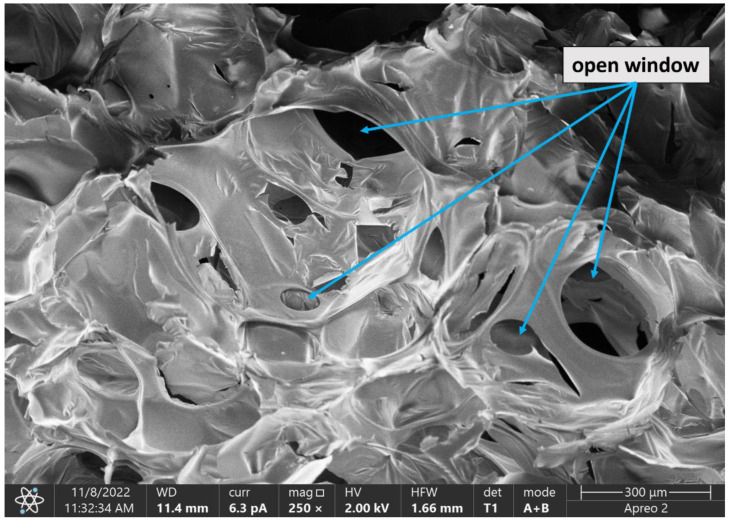
SEM image of the F_OR foam at a magnification of 250× with the open cells marked.

Figure 9 shows an SEM image of the F_OR foam set at a different angle from the foams in Figure 8 to better illustrate the continuity of open cells to see the gas virtual flow in the foam.

## 4. Conclusions

We demonstrate that, in addition to crude rapeseed oil and used rapeseed cooking oil, hemp oil and oilseed radish oil can also be sources of raw materials for the synthesis of bio-polyols for open-cell polyurethane foams. The synthesis method, which involves epoxidation of vegetable oils to an epoxy value of approximately 0.2 mol/100 g, allows obtaining bio-polyols with similar parameters regardless of the initial content of unsaturated bonds in the oils. The open-cell polyurethane foams produced in the experiment did not differ in terms of their apparent density, thermal conductivity, mechanical strength, water absorption and water vapour permeability. The properties of the foams were comparable to those of commercial products manufactured using petrochemical polyols, except for the higher apparent density of the foams based on bio-polyols by approximately 3–4 kg/m^3^. However, the bio-based foams had significantly lower water absorption, compared to polyurethane foams based on petrochemical polyols. Further research is required, especially to reduce the apparent density of bio-based open-cell foams.

## Figures and Tables

**Table 1:** Fatty acid content of the oils based on the literature references.

Common Name	Molecular Weight [g/mol]	C:U (p)	Fatty Acid Profile (%)		
			Rapeseed Oil ^a^ [29]	Oilseed Radish Oil [16]	Hemp Seed Oil ^b^ [23]
Myristic acid	228.4	14:0	0.1	-	-
Palmitic acid	256.4	16:0	4.5	6.13	7.76
Palmitoleic acid	254.4	16:1 (9)	0.4	-	0.16
Stearic acid	284.4	18:0	2.1	1.90	2.84
Oleic acid	282.4	18:1 (9)	64.5	23.87	11.95
Linoleic acid	280.4	18:2 (9, 12)	18.3	13.46	53.35
Linolenic acid	278.4	18:3 (9, 12, 15)	6.8	5.34	19.15
Arachidic acid	312.5	20:0	0.8	0.68	0.86
Gadoleic acid	310.5	20:1 (9)	1.3	8.58	-
Behenic acid	340.6	22:0	0.4	-	0.34
Erucic acid	338.6	22:1 (9)	0.8	31.76	0.07

Note: percentages may not add up to 100% due to presence of other minor fatty acids. C—number of carbon atoms; U—number of unsaturated bonds; p—position of the unsaturated bonds; ^a^—the same values can be assumed for UCO; ^b^—“Henola” variety.

**Table 2:** Formulation of PUR systems.

Components	Share, Parts by Weight
Bio-polyol	100
Polycat 15	2.2
Polycat 140	4.2
Tegostab B 8870	3.5
Dabco EM 400	3.5
Ortegol 500	0.6
TCPP	30
H_2_O	20
Isocyanate	203 ^a^

^a^ Calculated on the basis of polyol premix density of 1 g/cm^3^ and isocyanate density of 1.24 g/cm^3.^

**Table 3:** Selected properties of the vegetable oils used.

Oil	Ival [g I_2_/100 g]	Aval [mgKOH/g]	Viscosity [mPa∙s]	Mn [mol/100 g]	Mw [mol/100 g]	%H_2_O [wt %]
UCO	102.59 ± 0.67	2.88 ± 0.01	91 ± 5	847	850	0.14 ± 0.04
RO	105.78 ± 0.95	1.08 ± 0.01	88 ± 5	846	850	0.09 ± 0.02
OR	111.05 ± 0.56	4.24 ± 0.03	84 ± 5	836	841	0.07 ± 0.01
HSO	145.01 ± 1.29	4.40 ± 0.24	70 ± 5	836	841	0.11 ± 0.01

**Table 4:** Selected properties of the epoxidized oils.

Epoxidized Oil	Eval [mgKOH/g]	Ival [g I_2_/100 g]	Viscosity [mPa∙s]
E_UCO	0.187 ± 0.003	46.41 ± 0.64	110 ± 10
E_RO	0.196 ± 0.004	52.98 ± 0.82	120 ± 10
E_OR	0.189 ± 0.001	59.33 ± 0.46	130 ± 10
E_HSO	0.194 ± 0.002	93.27 ± 1.04	120 ± 10

**Table 5:** Selected properties of the bio-polyols

Epoxidized Oil	Hval [mgKOH/g]	Viscosity [mPa∙s]	Aval [mgKOH/g]	Mn [g/mol]	Mw [g/mol]	%H_2_O [wt.%]
P_UCO	169.6 ± 2.2	1430 ± 10	2.69 ± 0.03	1561	2786	0.21 ± 0.01
P_RO	177.8 ± 3.1	1620 ± 10	1.46 ± 0.01	1634	2950	0.11 ± 0.01
P_OR	171.7 ± 0.9	1230 ± 10	2.73 ± 0.03	1449	2455	0.14 ± 0.01
P_HSO	178.6 ± 1.6	1220 ± 10	3.31 ± 0.02	1437	2463	0.11 ± 0.02

**Table 6:** Properties of the polyurethane foams.

Foam	Apparent Density [kg/m^3^]	Thermal Conductivity [mW/m∙K]	Open-Cell Content [%]	Compressive Strength [kPa]	Water Vapour Permeability δ [mg/(m∙h∙Pa)]	Water Vapour Diffusion Resistance Factor μ [-]	Short-Term Water Absorption [kg/m^2^]	Cell Cross-Section Area [mm^2^]
F_UCO	11.2 ± 0.2	38.75 ± 0.85	97.71 ± 0.01	9.25 ± 1.80	0.15 ± 0.01	4.66 ± 0.17	0.18 ± 0.05	0.125 ± 0.082
F_RO	12.0 ± 0.1	36.10 ± 1.28	98.12 ± 1.26	12.63 ± 0.93	0.14 ± 0.01	5.23 ± 0.22	0.18 ± 0.05	0.077 ± 0.039
F_OR	12.1 ± 0.2	37.44 ± 1.27	97.42 ± 0.11	9.88 ± 0.57	0.14 ± 0.01	5.06 ± 0.19	0.25 ± 0.02	0.134 ± 0.912
F_HSO	11.2 ± 0.4	37.74 ± 0.86	98.58 ± 1.05	9.52 ± 2.04	0.12 ± 0.01	5.90 ± 0.63	0.21 ± 0.03	0.127 ± 0.086

## Data Availability

Not applicable.
